# The Benefits of Residual Hair Cell Function for Speech and Music Perception in Pediatric Bimodal Cochlear Implant Listeners

**DOI:** 10.1155/2018/4610592

**Published:** 2018-04-15

**Authors:** Xiaoting Cheng, Yangwenyi Liu, Bing Wang, Yasheng Yuan, John J. Galvin, Qian-Jie Fu, Yilai Shu, Bing Chen

**Affiliations:** ^1^Department of Otology and Skull Base Surgery, Eye and Ear, Nose, Throat Hospital of Fudan University, Shanghai, China; ^2^Key Laboratory of Hearing Medicine, National Health and Family Planning Commission, Shanghai, China; ^3^House Ear Institute, Los Angeles, CA, USA; ^4^Department of Head and Neck Surgery, David Geffen School of Medicine, UCLA, Los Angeles, CA, USA

## Abstract

**Objective:**

The aim of this study was to investigate the benefits of residual hair cell function for speech and music perception in bimodal pediatric Mandarin-speaking cochlear implant (CI) listeners.

**Design:**

Speech and music performance was measured in 35 Mandarin-speaking pediatric CI users for unilateral (CI-only) and bimodal listening. Mandarin speech perception was measured for vowels, consonants, lexical tones, and sentences in quiet. Music perception was measured for melodic contour identification (MCI).

**Results:**

Combined electric and acoustic hearing significantly improved MCI and Mandarin tone recognition performance, relative to CI-only performance. For MCI, performance was significantly better with bimodal listening for all semitone spacing conditions (*p* < 0.05 in all cases). For tone recognition, bimodal performance was significantly better only for tone 2 (rising; *p* < 0.05). There were no significant differences between CI-only and CI + HA for vowel, consonant, or sentence recognition.

**Conclusions:**

The results suggest that combined electric and acoustic hearing can significantly improve perception of music and Mandarin tones in pediatric Mandarin-speaking CI patients. Music and lexical tone perception depends strongly on pitch perception, and the contralateral acoustic hearing coming from residual hair cell function provided pitch cues that are generally not well preserved in electric hearing.

## 1. Introduction

For cochlear implant (CI) users, access to residual acoustic hearing in the contralateral ear can greatly benefit speech and music performance. Residual acoustic hearing provides detailed low-frequency information that can greatly benefit CI users under challenging listening condition. Bimodal listening—electric stimulation in one ear and acoustic stimulation (aided or unaided) in the contralateral ear—has been shown to significantly improve speech and music performance over the CI alone [1–26].

Most previous bimodal CI studies have been conducted with English-speaking CI users. For tonal languages such as Mandarin Chinese, the perception of lexical tones depends strongly on fundamental frequency (F0) cues [27]. The coarse spectral resolution provided by the CI is not sufficient to support complex pitch perception, which is needed for difficult listening tasks such as music perception, F0 perception, and speech understanding in noise [28]. Despite the weak F0 cues, Mandarin-speaking CI users are able to achieve moderately good tone recognition performance [20, 29–34], most likely due to perception of amplitude contour and duration cues that covary with F0 in naturally uttered Chinese tones [29, 34]. For patients with some amount of residual acoustic hearing, combining a hearing aid (HA) with the CI may represent the best opportunity to improve CI users' Chinese tone recognition. Aided acoustic hearing may provide the F0 cues necessary for tone recognition in addition to amplitude and duration cues available with the CI.

Previous studies with Chinese-speaking CI users have shown significant benefits for bimodal listening over the CI alone. Yuen et al. [24] measured tone and disyllable word recognition in quiet and in noise in 15 Mandarin-speaking pediatric bimodal CI listeners aged 5 to 14 years old. Head shadow benefits in noise, tone, and disyllable word recognition were significantly better with bimodal than CI-only listening. Li et al. [35] found significantly better tone, vowel, and consonant recognition with bimodal listening (relative to CI-only) in 12 Mandarin-speaking CI users aged 16 to 24 years old. Interestingly, the bimodal benefit for tone recognition in quiet was significantly correlated with CI experience, suggesting that bimodal CI users learn to better combine the low-frequency spectrotemporal cues from acoustic hearing with the temporal envelope cues from electric hearing over time. Chang et al. [36] measured Mandarin tone, syllable, and vowel recognition in 15 prelingually deaf Mandarin-speaking bimodal CI users aged 10 to 20 years old. Tone and syllable recognition significantly improved with bimodal listening, while vowel recognition did not. Correlation analyses revealed that the bimodal benefits could not be predicted by acoustic hearing thresholds in the nonimplanted ear or by demographic variables of the participants. Yang and Zeng [37] measured bimodal benefits in 13 Mandarin-speaking bimodal listeners aged 5 to 46 years old (mean: 15.3 years old). There was a significant bimodal benefit for word recognition, largely due to better consonant and tone recognition.

Taken together, these previous studies demonstrated bimodal benefits in adult and pediatric Mandarin-speaking CI users for some listening tasks and conditions. However, the bimodal benefits varied across studies, and the number of subjects in each study was relatively small (4–15 subjects, depending on the study and conditions). Previous studies have also shown that Mandarin-speaking pediatric CI users have difficulty with pitch-related music perception, such as melodic contour identification (MCI; [20]). Crew et al. [5] showed that combined acoustic and electric hearing provides significantly better MCI recognition performance when comparing to CI-only conditions in English-speaking CI adults. Mandarin-speaking pediatric CI users may similarly benefit from combined acoustic and electric hearing for melodic pitch perception, but this has yet to be tested.

In this study, bimodal benefits for music and speech perception were studied in a large cohort of Mandarin-speaking pediatric CI users (*n* = 35). Music perception was measured using an MCI task, and Mandarin speech perception in quiet was measured using vowel, consonant, tone, and sentence recognition tasks. Performance was measured with the CI-only or with the CI + HA. Bimodal and CI-only performances were compared to various demographic variables, and music and speech perception was compared to one another to observe potential contributions of pitch cues to the different listening tasks.

## 2. Materials and Methods

### 2.1. Ethics Statement

The study and the informed consent procedures were approved by the local ethics committee (Ethics Committee of the Eye, Ear, Nose, and Throat Hospital, Fudan University, approval number: KY2012-009), and written informed consent was obtained from children's parents before participation.

### 2.2. Subjects

Thirty-five (10 females and 25 males) Mandarin-speaking pediatric CI patients were recruited from the Shanghai Rehabilitation Center, Shanghai, China. The inclusion criteria were that all pediatric participants used a CI in one ear and a HA in the contralateral ear for at least 6 months. The exclusion criteria were formal music training experience, as well as any cognitive, visual, and intelligence disorders. Across all CI subjects, the mean age at testing was 6.5 years (range: 4.9–12.3 years), the mean age at implantation was 2.9 years (range: 0.9–7.0 years), the mean CI experience was 3.5 years (range: 0.6–8.1 years), and the mean HA experience was 2.7 years (range: 0.5–9.0 years). Demographic information is shown in Table 1.

### 2.3. Audiometric Thresholds

Aided thresholds with the CI-only and the HA-only were measured in sound field using warble tones and using subjects' clinical settings for the CI and HA. All subjects were tested in a sound-treated booth and seated directly facing a single loudspeaker positioned 1 m away from the subject. Unaided thresholds were collected using pure tone with headphones. Pure-tone average (PTA) thresholds across 0.5, 1.0, and 2.0 kHz are shown for each subject in Table 1.

### 2.4. Music and Speech Perception

All stimuli were presented in sound field at 65 dBA. Music and speech perception was measured with the CI-only and with the CI + HA; subjects were tested using the clinical settings for each device, which were not changed throughout the study. All stimuli were presented, and responses were collected using custom software (Mandarin Angel Sound software; freely available at http://mast.emilyfufoundation.org); performance was scored in terms of percent correct.

#### 2.4.1. Music Stimuli and Test Procedures

MCI stimuli were similar to those in previous studies (Galvin et al. [38, 39]) and consisted of nine melodic contours (rising, rising-flat, rising-falling, flat-rising, flat, flat-falling, falling-rising, falling-flat, or falling), composed of five notes of equal duration (250 ms, with 50 ms of silence between each note). The lowest note in any contour was C4 (262 Hz). The spacing between successive notes in each contour was varied to be 1, 2, 3, or 5 semitones. The instrument used for the contour was a piano sample, as in Galvin et al. [39]. Thus, the stimulus set consisted of 36 stimuli (9 melodic contours × 4 semitone spacing), and all 36 stimuli were presented during each test run.

MCI was measured using a 9-alternative forced choice (9-AFC) procedure. Prior to formal testing, a practice session was conducted to familiarize subjects with the stimuli, task, and procedures. During testing, a contour would be randomly selected from the stimulus set and presented to the subject, who responded by clicking on one of the response boxes shown on the computer screen.

#### 2.4.2. Mandarin Tone Recognition in Quiet

Mandarin tone stimuli consisted of 4 tonal patterns produced by two males and two females, taken from the Standard Chinese Database recorded at University of Science and Technology of China [40]. The four tonal patterns included tone 1 (high-level), tone 2 (high-rising), tone 3 (falling-rising), and tone 4 (high-falling), produced for 4 monosyllables (b/a/, b/o/, b/u/, and b/i/). Thus, the stimulus set consisted of 64 stimuli (4 tones × 4 monosyllables × 4 talkers), and all 64 stimuli were presented during each test run. During testing, a stimulus would be randomly selected from the stimulus set and presented to the subject, who responded by clicking on one of the 4 response boxes (labelled according to tone number) shown on the computer screen. No trial-by-trial feedback or training was provided.

#### 2.4.3. Vowel Recognition in Quiet

Vowel stimuli were monosyllabic words produced by one male and one female talker, taken from the same Standard Chinese Database as tone stimuli. Vowel stimuli consisted of six groups of 4 vowels each; the initial consonant for each group was the same. The six groups of vowel stimuli included (1) yá, yáng, yú, yíng, (2) mò, mù, mèi, miè, (3) qiú, qué, qín, qún, (4) guī, gōu, gēn, gōng, (5) shé, shí, sháo, shéng, and (6) chá, chái, chán, chún. Thus, there were 24 vowel stimuli in the stimulus set. During testing, a group would be randomly selected, and a vowel stimulus would be randomly selected from within the group and presented to the subject, who responded by clicking on one of the 4 response choices labelled according to the vowels in the selected group. No trial-by-trial feedback or training was provided. All 24 stimuli were presented during the test run.

#### 2.4.4. Consonant Recognition in Quiet

Consonant stimuli were monosyllabic words produced by one male and one female talker, taken from the same Standard Chinese Database as tone stimuli. Similar to the vowel stimuli, consonant stimuli consisted of six groups of 4 consonants each; the final vowel for each group was the same. The six groups of consonant stimuli included (1) jì, rì, cì, sì, (2) pí, lí, qí, xí, (3) fù, tù, nù, bù, (4) gŭ, hŭ, zhŭ, wŭ, (5) gŏu, kŏu, shŏu, zŏu, and (6) māo, dāo, chāo, yāo. Thus, there were 24 consonant stimuli in the stimulus set. During testing, a group would be randomly selected, and a consonant stimulus would be randomly selected from within the group and presented to the subject, who responded by clicking on one of the 4 response choices labelled according to the consonants in the selected group. No trial-by-trial feedback or training was provided. All 24 stimuli were presented during the test run.

#### 2.4.5. Sentence Recognition in Quiet

Sentence recognition was measured using sentences from the Mandarin speech perception (MSP) test, which consisted of 10 lists of 10 sentences, each sentence with 7 syllables [41, 42]. Sentence recognition was measured using an open-set paradigm. During testing, a list was randomly selected, and a sentence was randomly selected from the list and presented to the subject, who repeated as many words as possible. The experimenter scored the correctly identified words. One MSP list was presented for each test session, and no lists were repeated within test subjects.

## 3. Results

**Figure 1:**
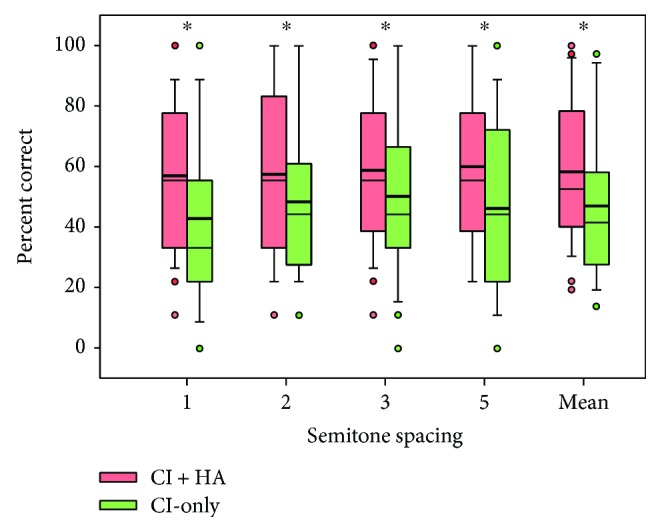
Box plots of MCI scores for different semitone spacing and across all semitone spacing with the CI-only and with the CI + HA. The boxes show the 25th and 75th percentiles, the error bars show the 5th and 95th percentiles, the circles show outliers, the thin solid line shows the median, the thick solid line shows the mean, and the asterisks indicate significant differences between CI + HA and CI-only performance.

Figure 1 shows boxplots of MCI scores with the CI-only and with CI + HA. Mean MCI performance improved from 47% correct with the CI-only to 58% correct with CI + HA. A two-way RM ANOVA with listening condition (CI, CI + HA) and semitone spacing (1, 2, 3, and 5) as factors showed a significant effect for listening condition [*F*(1,102) = 30.9, *p* < 0.001], but not for semitone spacing [*F*(3,102) = 2.2, *p* = 0.098]; there was no significant interaction [*F*(3,102) = 0.9, *p* = 0.427]. Post hoc Bonferroni pairwise comparisons showed that MCI performance was significantly better with CI + HA than with the CI-only for all semitone spacing conditions (*p* > 0.05 in all cases).

**Figure 2:**
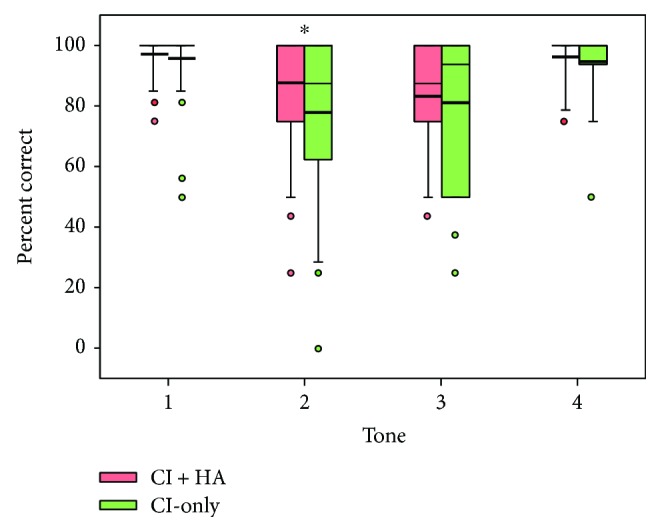
Box plots of tone recognition scores for the different lexical tones with the CI-only and with the CI + HA. The boxes show the 25th and 75th percentiles, the error bars show the 5th and 95th percentiles, the circles show outliers, the thin solid line shows the median, the thick solid line shows the mean, and the asterisks indicate significant differences between CI + HA and CI-only performance.

Figure 2 shows boxplots of tone recognition scores with the CI-only and with CI + HA. A two-way RM ANOVA with listening condition and lexical tone (1, 2, 3, and 4) as factors showed significant effects for listening condition [*F*(1,102) = 4.9, *p* = 0.034] and lexical tone [*F*(3,102) = 11.9, *p* < 0.001]; there was a significant interaction [*F*(3,102) = 3.2, *p* = 0.028]. Post hoc Bonferroni pairwise comparisons showed that performance was significantly better with CI + HA only for tone 2 (*p* < 0.05). With the CI + HA, performance was significantly poorer with tone 3 than with tone 1 or tone 4 (*p* < 0.05 in both cases). With the CI-only, performance was significantly better tones 1 and 4 than with tones 2 and 3 (*p* < 0.05 in all cases).

**Figure 3:**
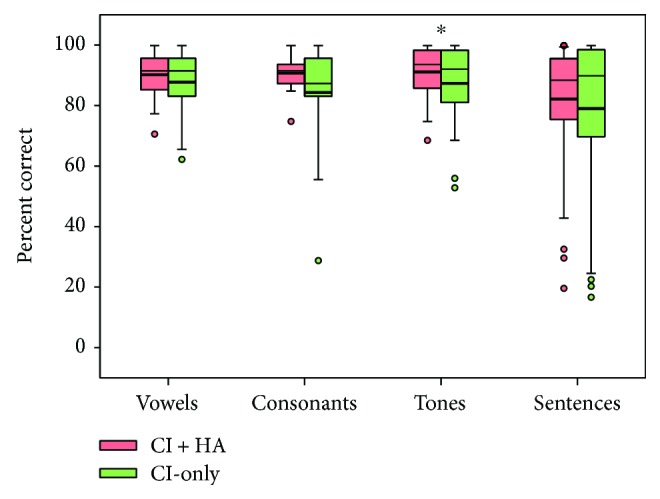
Box plots of vowel, consonant, tone (averaged across all 4 lexical tones), and sentence recognition scores with the CI-only and with the CI + HA. The boxes show the 25th and 75th percentiles, the error bars show the 5th and 95th percentiles, the circles show outliers, the thin solid line shows the median, the thick solid line shows the mean, and the asterisks indicate significant differences between CI + HA and CI-only performance.

Figure 3 shows boxplots of vowel, consonant, tone, and sentence recognition scores with the CI-only and CI + HA. Note that due to time constraints, vowel and consonant recognition was measured in only 17 subjects; tone and sentence recognition was measured in all 35 subjects. Mean vowel recognition improved from 88% correct with the CI-only to 90% correct with CI + HA. A one-way RM ANOVA showed no significant difference between CI-only and CI + HA [*F*(1,16) = 1.1, *p* = 0.302]. Mean consonant recognition improved from 84% correct with the CI-only to 91% correct with CI + HA. A one-way RM ANOVA showed no significant difference between CI-only and CI + HA [*F*(1,16) = 3.0, *p* = 0.103]. Mean tone recognition improved from 87% correct with the CI-only to 91% correct with CI + HA. A one-way RM ANOVA showed that performance was significantly better with the CI + HA than with the CI-only [*F*(1,34) = 4.9, *p* = 0.033]. Mean sentence recognition improved from 79% correct with the CI-only to 82% correct with CI + HA. A one-way RM ANOVA showed no significant difference between CI-only and CI + HA [*F*(1,34) = 1.7, *p* = 0.203].

Demographic variables age at testing, age at cochlear implantation, duration of deafness, CI experience, HA experience, aided PTA threshold, and unaided PTA thresholds were compared to MCI, vowel recognition, consonant recognition, mean tone recognition, and sentence recognition with the CI-only or the CI + HA using Pearson correlations. Note that for the correlations for MCI, tone, and sentence recognition, *n* = 35; for vowel and consonant recognition, *n* = 17. The results are shown in Table 2. There were no significant correlations between any of the demographic variables and MCI or vowel recognition performance with the CI-only or with the CI + HA (*p* > 0.05 in all cases). Consonant recognition with the CI-only or with the CI + HA was negatively correlated with age at CI and duration of deafness (*p* < 0.05 in all cases); consonant recognition with the CI-only was also correlated with CI experience (*p* < 0.05). Tone recognition with the CI-only or with the CI + HA was correlated with CI experience (*p* < 0.05 in both cases); tone recognition with the CI + HA was negatively correlated with age at cochlear implantation (*p* < 0.05). Sentence recognition with the CI-only was negatively correlated with age at cochlear implantation and duration of deafness (*p* < 0.05 in both cases) and correlated with CI experience and bimodal experience (*p* < 0.05 in both cases). Sentence recognition with the CI + HA was correlated with unaided PTA thresholds (*p* < 0.05).

Pearson correlation analyses were also performed among the various music and speech tests. With the CI-only or with the CI + HA, there were no significant correlations between MCI and any of the speech tests (*p* > 0.05 in all cases). With the CI-only or with the CI + HA, there were significant correlations among all the speech tests (*p* < 0.05 in all cases).

## 4. Discussion

The present data show that combined acoustic and electric hearing can significantly improve Mandarin-speaking pediatric CI patients' music and Mandarin tone perception, two listening tasks in which pitch cues are important. However, there was no significant bimodal benefit for vowel, consonant, or sentence recognition in quiet. Speech performance with the CI-only or with the CI + HA was significantly correlated with age at implantation and duration of deafness, underscoring the benefit of early implantation. Tone recognition was significantly correlated with all other speech measures, underscoring the strong contribution of lexical tone perception to Mandarin speech perception. Below, we discuss the results in greater detail.

### 4.1. CI-Only Music and Speech Performance

#### 4.1.1. Music Perception

Mean MCI performance was generally poor (47% correct) and highly variable (range: 17–97% correct). Mean MCI performance was significantly better (*p* < 0.001) than the 23% correct reported in Tao et al. [20], but comparable (*p* = 0.11) to the 34% correct reported in Fu et al. [43]; both studies were conducted with Chinese CI users. CI-only performance was also comparable to that in previous studies with adult English-speaking CI users [6, 38, 39]. In this study, there was no significant effect of semitone spacing, consistent with Tao et al. [20], who showed no significant differences among semitone spacing, except for between 1 and 6 semitones. Differences in subject age, duration of deafness, and previous acoustic hearing experience may have also contributed to differences in MCI performance observed between this and previous studies.

#### 4.1.2. Speech Perception

Mean tone recognition with the CI-only was 87% correct, and recognition of tones 2 and 3 was significantly poorer than recognition of tones 1 and 4. While mean tone recognition score was comparable to the 81% correct reported in Tao et al. [20], recognition of individual tones differed between these studies even though the test materials and procedures were exactly the same. In Tao et al. [20], recognition of tone 2 was significantly poorer than that of tones 1, 3, and 4, and recognition of tone 4 was significantly better than that of the tones 1, 2, and 3. Recognition of tone 1 in this study was significantly poorer than that of Tao et al. [20] (*p* < 0.05), with no significant difference in between studies in recognition of tones 2, 3, and 4. Note that while significant, performance differences were generally small across these studies.

Mean baseline MSP sentence recognition was 79% correct, comparable to the 85% correct reported by Su et al. [44] for pediatric CI patients, but much higher than the 59% correct reported by Li et al. [45] for adult CI patients. Mean vowel (87% correct) and consonant (84% correct) recognition scores in quiet were much higher than reported in Li et al. [45] (58.9% and 45.8% correct for vowels and consonants, resp.). It is possible that differences in age at testing and duration of deafness may have contributed to the discrepancies in sentence recognition across studies. In Li et al. [45], adult subjects were tested; in China, adult CI users often experience a longer duration of hearing loss before implantation than children. Note also that phoneme recognition was measured using a 20-AFC procedure in Li et al. [45], compared to the 4-AFC procedure with multiple subsets of stimuli in this study.

### 4.2. Bimodal Benefits for Music and Speech Perception

#### 4.2.1. Music Perception

Relative to CI-only, bimodal MCI performance improved by 11 percentage points. Mean bimodal MCI performance (58% correct) was poorer than the 72% correct reported for English-speaking adult bimodal listeners in Crew et al. [6]; in both studies, CI-only performance was comparable. Interestingly, bimodal performance was slightly poorer than HA-only performance in Crew et al. [6], suggesting that there was little bimodal benefit over the HA alone. In this study, HA-only performance was not measured. It is possible that the HA may have similarly carried MCI perception with bimodal listening; if so, it is unclear whether the present bimodal subjects experienced interference between acoustic and electric hearing. Alternatively, performance with the HA might have been poorer than that observed in Crew et al. [6]. Note that a slightly higher base note (the lowest note in a contour) was used in this study (C4 or 262 Hz) than in Crew et al. [5] (A3 or 220 Hz). Depending on the amount of aided acoustic hearing, some notes in the contours may have been near the limits of aided acoustic hearing. Finally, differences between postlingual adults in Crew et al. [6] and the present prelingual pediatric CI users may have contributed to differences in bimodal MCI performance.

Bimodal MCI performance was significantly better than CI-only performance, in agreement with previous studies that showed a bimodal advantage for music perception [5, 10, 11, 13, 19, 46, 47]. Previous studies have shown that adding low-frequency acoustic hearing in the contralateral ear can improve CI users' pitch perception ([46, 48, 49]). Chen et al. [48] also found a significant correlation between HA experience and bimodal pitch perception in pediatric CI users, suggesting that HA experience before and/or implantation may help to develop pitch pattern perception. However, other studies have not shown significant bimodal advantages for music perception. Prentiss et al. [50] found a significant bimodal advantage for music chord perception, but not for musical timbre perception. Bartov and Most [51] found a bimodal advantage for song identification when listeners were presented with simple, tonal representations, but not for full arrangements, a cappella versions, or melodic and rhythmic versions. Thus, bimodal benefits may differ according to the amount of acoustic hearing in the contralateral ear, the amount of HA and/or bimodal listening experience, subject age, status of hearing loss (prelingual or postlingual), and the musical listening task.

#### 4.2.2. Speech Perception

The present results showed a small but significant bimodal benefit for tone recognition (largely due to improved recognition of tone 2), consistent with previous findings ([35, 36]). However, there was no significant bimodal benefit for vowel, consonant, or sentence recognition in quiet, consistent with some previous studies [18, 35]. Li et al. [35] found a significant bimodal benefit for vowel recognition in quiet in adult Mandarin-speaking CI users, but not for tone or vowel recognition in quiet. Rathna-Kumar et al. [18] found a bimodal benefit for speech understanding in noise in India-speaking pediatric CI users, but not for speech understanding in quiet. Note that the variability in performance was reduced with the CI + HA, relative to CI-only.

One limit for bimodal benefits may have been ceiling performance for the speech perception measures in quiet. With the CI-only, mean tone, vowel, consonant, and sentence recognition performance was 87.3%, 87.7%, 84.3%, and 79.4% correct, respectively. With the CI + HA, mean tone, vowel, consonant, and sentence recognition performance improved by 3.8, 2.4, 6.4, and 3.1 percentage points, respectively. Most previous studies have shown bimodal benefits for speech understanding in noise (e.g., [3, 5, 6, 8, 9, 13, 18, 25, 26]). Although HA-only performance was not measured in the present study, there was likely a strong performance asymmetry between the HA and CI ears in the present subjects. Yoon et al. [23] showed a greater bimodal benefit when the performance asymmetry between ears was reduced. While it is likely that the present group of prelingual Mandarin pediatric CI users might have received a bimodal benefit in noise, this should be tested in a similarly large cohort.

### 4.3. Correlational Analyses

With the CI-only, consonant, tone, and sentence recognition was significantly correlated with CI experience. Consonant and sentence recognition was negatively correlated with age at implantation and duration of deafness. Taken together, these correlations underscore the benefit of early implantation for pediatric CI users. Interestingly, CI-only sentence recognition was significantly correlated with bimodal listening experience. It is possible that previous acoustic hearing or listening with the combined acoustic and electric hearing may have strengthened CI-only speech pattern recognition performance.

With the CI + HA, consonant and tone recognition was significantly correlated with age at implantation, and consonant recognition was significantly correlated with duration of deafness. While there were no significant correlations between sentence recognition and age at implantation (*r* = −0.32; *p* = 0.058), duration of deafness (*r* = −0.33; *p* = 0.056), CI experience (*r* = 0.30; *p* = 0.077), or HA experience (*r* = 0.30; *p* = 0.079), the relationship between sentence recognition and these demographic variables approached significance. Interestingly, unaided (rather than aided) PTA thresholds were significantly correlated with bimodal sentence recognition. The unaided PTA thresholds may reflect (to some degree) the health of the nonimplanted ear, with higher thresholds indicating poorer nerve survival. Aiding better ears may have required less amplification, compression, and overall signal distortion; broader auditory filters with greater hearing loss may have exacerbated distortion to the signal associated with the HA processing.

Significant correlations were observed among vowel, consonant, tone, and sentence recognition with the CI-only and with CI + HA, underscoring the importance of tone perception for sentence recognition [52]. Somewhat surprisingly, there were no correlations between MCI and any speech performance measures with the CI-only or with CI + HA. Given that pitch cues are important for both listening tasks, one might expect that better pitch perception would benefit both MCI and tone recognition. Tao et al. [20] also found no significant correlation between MCI and tone recognition in young Mandarin-speaking CI users. In both studies, ceiling performance for tone recognition in quiet most likely limited correlations. Tone recognition in noise might reduce ceiling performance effects and possibly show a relationship between MCI and tone recognition. Note that pitch cues in the MCI task occurred within a 1500 ms contour, while pitch cues for tone recognition occurred within a 300 ms syllable. Also, CI users were able to make use of duration and amplitude cues for tone recognition which may have contributed to ceiling performance effects; for MCI, duration and amplitude cues were kept constant within the contours.

## 5. Conclusions

Music and Mandarin speech perception was measured in 35 pediatric Chinese CI users with the CI alone and with the CI + HA (bimodal listening). Key findings include the following:

1. Performance was significantly better with bimodal listening than with the CI-only for MCI and tone perception in quiet. There was no significant bimodal advantage for vowel, consonant, or sentence recognition in quiet.
2. With the CI-only, significant correlations were observed between CI experience and consonant, tone, and sentence recognition, between age at implantation and consonant and tone recognition, and between duration of deafness and consonant and tone recognition, underscoring the benefit of early implantation for Mandarin-speaking pediatric CI users.
3. With the CI + HA, significant correlations were observed between age at implantation and consonant and tone recognition and between duration of deafness and consonant recognition. While not significant, notable relationships were observed between sentence recognition and age at implantation, duration of deafness, CI experience, and HA experience, suggesting that early implantation may benefit combined acoustic and electric hearing.
4. There were significant correlations among all speech measures, underscoring the importance of tone perception to Mandarin sentence recognition. Despite the importance of pitch cues to both listening tasks, there was no correlation between MCI and tone recognition, most likely due to ceiling performance effects associated with tone recognition in quiet.

## Figures and Tables

**Table 1:** CI subject demographic information.

Subject	Etiology	Gender	Age at testing (yrs)	Age at CI (yrs)	Dur deaf (yrs)	CI exp (yrs)	HA exp (yrs)	CI device	CI strategy	Aided PTA dB HL	Unaided PTA dB HL
S1	Unknown	F	9.2	4.0	4.0	5.2	1.00	AB HiRes 90K	F120	36.7	70.0
S2	Unknown	M	7.3	3.1	3.1	4.2	3.80	Cochlear N-24	ACE	45.0	70.0
S3	LVAS	F	5.0	3.0	2.0	1.0	2.00	Cochlear N-24	ACE	40.0	73.3
S4	Unknown	M	10.0	5.0	5.0	5.0	9.00	Cochlear N-24	ACE	53.3	73.3
S5	Unknown	M	5.9	1.3	1.3	4.6	4.00	AB HiRes 90K	F120	33.3	75.0
S6	LVAS	F	8.0	2.3	2.3	5.7	6.00	MED-EL Pulsar	FSP	38.3	80.0
S7	LVAS	F	5.0	2.0	3.0	3.0	3.00	MED-EL Pulsar	FSP	40.0	83.3
S8	LVAS	M	7.2	5	2	2.2	2.00	AB HiRes 90K	F120	41.7	83.3
S9	Unknown	M	5.8	1.0	1.0	4.8	5.00	Cochlear N-24	ACE	35.0	88.3
S10	LVAS	M	6	1.5	1.5	4.5	3.30	AB HiRes 90K	F120	46.7	88.3
S11	LVAS	M	5.3	4.7	4.7	0.6	4.70	AB HiRes 90K	F120	35.0	90.0
S12	Unknown	M	8.1	7.0	2.0	1.1	3.00	MED-EL Pulsar	FSP	38.3	90.0
S13	LVAS	F	6.1	3.5	1.2	2.6	3.80	MED-EL Pulsar	FSP	48.3	91.7
S14	Unknown	M	5.9	3.1	3.1	2.8	1.00	MED-EL Pulsar	FSP	45.0	93.3
S15	LVAS	M	5.5	2	1	3.5	4.50	MED-EL Pulsar	FSP	40.0	96.7
S16	Unknown	F	5.6	2.1	2.1	3.5	2.00	Cochlear N-24	ACE	46.7	96.7
S17	Unknown	M	6.5	2.3	2.3	4.2	0.50	Cochlear N-24	ACE	53.3	96.7
S18	Unknown	F	5.2	1	1	4.2	1.00	MED-EL Pulsar	FSP	45.0	98.3
S19	Unknown	M	12.3	5.6	5.6	6.7	2.50	Cochlear N-24	ACE	56.7	98.3
S20	Unknown	M	5.8	3.3	3.3	2.5	1.00	Cochlear N-24	ACE	50.0	98.3
S21	Unknown	M	5.2	2.8	2.8	2.4	1.00	MED-EL Pulsar	FSP	36.7	98.3
S22	Unknown	M	5.2	3.1	3.1	2.1	5.00	MED-EL Pulsar	FSP	63.3	100.0
S23	LVAS	M	8.4	3.1	3.1	5.3	0.50	AB HiRes 90K	F120	56.7	105.0
S24	Unknown	F	5	1	4	3	4.00	Cochlear N-24	ACE	56.7	105.0
S25	Unknown	F	7.0	6.0	6.0	1.0	3.00	MED-EL Pulsar	FSP	58.3	106.7
S26	Unknown	M	5.2	1	1	4.2	0.60	MED-EL Pulsar	FSP	75.0	108.3
S27	Unknown	M	10.0	1.9	1.9	8.1	7.00	Cochlear N-24	ACE	70.0	108.3
S28	Unknown	M	5.2	3.0	3.0	2.2	1.50	Cochlear N-24	ACE	46.7	108.3
S29	Unknown	M	4.9	0.9	0.9	4	4.00	Cochlear N-24	ACE	65.0	110.0
S30	Unknown	M	6	2.6	2.6	3.4	1.00	MED-EL Pulsar	FSP	46.7	110.0
S31	Unknown	M	5.9	3.4	3.4	2.5	1.00	Cochlear N-24	ACE	60.0	110.0
S32	Unknown	M	6.8	2	2	4.8	1.00	Cochlear N-24	ACE	63.3	111.7
S33	Unknown	F	7.8	5.8	5.8	2.0	0.50	Cochlear N-24	ACE	61.7	111.7
S34	Unknown	M	5.1	0.9	0.9	4.2	1.00	MED-EL Pulsar	FSP	95.0	115.0
S35	Unknown	M	5.0	4.0	4.0	5.2	1.00	Cochlear N-24	ACE	66.7	115.0
AVE			6.5	2.9	2.7	3.5	2.7			51.1	96.0
SE			0.3	0.3	0.2	0.3	0.4			2.3	2.3

LAVS = large vestibular aqueduct syndrome; F = female; M = male; age at CI = age at cochlear implantation; dur deaf = duration of deafness; CI exp = CI experience; HA exp = HA experience; N-24 = Nucleus 24; AB = Advanced Bionics; ACE = advanced combination encoder; F120 = Fidelity 120; FSP=fine-structure processing; PTA = pure-tone average threshold across 0.5, 1.0, and 2.0 kHz. AVE = average; SE = standard error.

**Table 2:** Pearson correlations between demographic variables and music and speech perception.

		MCI		Vowel		Consonant		Tone		Sentence	
		*r*	*p*	*r*	*p*	*r*	*p*	*r*	*p*	*r*	*p*
CI-only	Age test	−0.02	0.904	−0.05	0.763	0.14	0.581	0.38	0.137	0.04	0.827
	Age CI	−0.08	0.669	−0.25	0.148	−0.50	*0.039* ^∗^	−0.36	0.153	−0.43	*0.009* ^∗^
	Dur deaf	−0.06	0.735	−0.32	0.059	−0.50	*0.039* ^∗^	−0.32	0.208	−0.44	*0.008* ^∗^
	CI exp	0.03	0.863	0.17	0.327	0.55	*0.023* ^∗^	0.70	*0.002* ^∗^	0.44	*0.008* ^∗^
	HA exp	0.13	0.470	−0.07	0.686	−0.10	0.697	−0.05	0.848	0.20	0.239
	Bimodal exp	0.14	0.428	0.09	0.615	0.27	0.301	0.43	0.0878	0.38	*0.024* ^∗^
	Aided PTA	0.17	0.351	0.17	0.522	0.24	0.362	0.09	0.603	0.01	0.977
	Unaided PTA	−0.02	0.911	−0.13	0.454	−0.19	0.476	−0.10	0.693	−0.33	0.055

CI + HA	Age test	−0.04	0.807	−0.14	0.440	−0.12	0.635	0.15	0.563	<0.01	0.997
	Age CI	−0.05	0.796	−0.21	0.234	−0.67	*0.003* ^∗^	−0.50	*0.039* ^∗^	−0.32	0.058
	Dur deaf	−0.18	0.327	−0.26	0.138	−0.66	*0.004* ^∗^	−0.41	0.099	−0.33	0.056
	CI exp	<0.01	0.984	0.06	0.729	0.43	0.083	0.58	*0.014* ^∗^	0.30	0.077
	HA exp	0.26	0.148	0.00	0.980	0.23	0.376	0.17	0.519	0.30	0.079
	Bimodal exp	0.23	0.206	0.06	0.752	0.37	0.149	0.43	0.087	0.3	0.082
	Aided PTA	0.02	0.914	−0.08	0.635	0.07	0.785	−0.02	0.932	−0.08	0.645
	Unaided PTA	−0.11	0.529	−0.15	0.399	0.13	0.618	−0.09	0.735	−0.39	*0.023* ^∗^

The italics and asterisks indicate significant correlations (*p* < 0.05). MCI = melodic contour identification; age test = age at testing; age CI = age at cochlear implantation; dur deaf = duration of deafness; CI exp = cochlear implant experience; HA exp = hearing aid experience; PTA = pure-tone average threshold.
